# Educational differences in the prevalence of behavioural risk factors in Germany and the EU – Results from the European Health Interview Survey (EHIS) 2

**DOI:** 10.25646/6225

**Published:** 2019-12-11

**Authors:** Jonas D. Finger, Jens Hoebel, Benjamin Kuntz, Ronny Kuhnert, Johannes Zeiher, Gert B. M. Mensink, Thomas Lampert

**Affiliations:** 1 Formerly Robert Koch Institute, Berlin Department of Epidemiology and Health Monitoring; 2 Robert Koch Institute, Berlin Department of Epidemiology and Health Monitoring

**Keywords:** HEALTH BEHAVIOUR, EDUCATIONAL DIFFERENCES, ADULTS, GERMANY, EUROPEAN COMPARISON

## Abstract

This article examines educational differences in the prevalence of behavioural risk factors among adults and compares the results for Germany with the average from the European Union (EU). Data were derived from the second wave of the European Health Interview Survey, which took place between 2013 and 2015 (EHIS 2). Analyses were conducted using a regression-based calculation of relative and absolute educational differences in the prevalence of behavioural risk factors, based on self-reported data from women and men aged between 25 and 69 (n=217,215). Current smoking, obesity, physical activity lasting less than 150 minutes per week, heavy episodic drinking and non-daily fruit or vegetable intake are more prevalent among people with a low education level than those with a high education level. This applies to Germany as well as the EU average. Overall, the relative educational differences identified for these risk factors place Germany in the mid-range compared to the EU average. However, relative educational differences in current smoking and heavy episodic drinking are more manifest among women in Germany than the EU average, with the same applying to low physical activity among men. In contrast, relative educational differences in non-daily fruit or vegetable intake are less pronounced among women and men in Germany than the average across the EU. Increased efforts are needed in various policy fields to improve the structural conditions underlying health behaviour, particularly for socially disadvantaged groups, and increase health equity.

## 1. Introduction

Considerable social differences exist with regard to mortality in Germany and other European Union (EU) Member States, with socially disadvantaged groups being at higher risk of premature death than those who are better off [1, 2]. Furthermore, although there is evidence that absolute inequalities in mortality between socioeconomic groups have decreased since the 1990s, relative inequalities have continued to increase in some European countries [2]. Non-communicable diseases (NCDs) such as cardiovascular diseases, cancers, chronic obstructive pulmonary disease (COPD) and diabetes mellitus account for approximately 90% of deaths and 84% of the disease burden in Europe; such diseases also have a negative impact on the general well-being of the population and pose a challenge to health systems and economic development [3]. According to calculations made for the 2017 Global Burden of Disease Study, coronary heart disease (CHD) continues to be the leading cause of death in Western Europe, accounting for 15.9% of total mortality. Furthermore, CHD also has the greatest impact on developments in social inequalities in mortality in Europe [2, 4]. The governments of EU Member States have therefore set themselves the goal of reducing premature deaths from NCD by one third (compared to 2010 levels) by 2030 [5]. Behavioural risk factors are partially responsible for many of these deaths. Attributable risk is a means of identifying the proportion of deaths that are associated with a particular risk factor. The attributable risk associated with deaths from CHD has been calculated at 11.4% for low vegetable intake, 7.3% for low fruit intake, 14.2% for smoking, 11.7% for low physical activity and 17.5% for a high body mass index (BMI) [4]. Smoking has been shown to be responsible for 70.4% of lung cancers and 44.3% of deaths due to COPD [4]. High alcohol intake has been shown to be associated with a 20.2% attributable risk of bowel cancer [4]. A recent European study, which was based on the pooled data set used by the 2014 European Social Survey gained and collected from 21 European countries, found considerable educational differences in risky health behaviour in Europe [6].

**GEDA 2014/2015-EHIS (for international comparisons)**

**Data holder:** Robert Koch Institute

**Aims:** To provide reliable information about the population’s health status, health behaviour and health care in Germany, with the possibility of a European comparison

**Method:** Questionnaires completed on paper or online

**Population:** People aged 15 years and above with permanent residency in Germany

**Sampling:** Registry office sample; randomly selected individuals from 301 communities in Germany were invited to participate

**Participants:** 24,824 people (13,568 women, 11,256 men)

**Response rate:** 27.6%

**Study period:** November 2014 - July 2015

More information in German is available at www.geda-studie.de and Lange et al. 2017 [9]

From a health-policy perspective, it is therefore essential that social differences be analysed with regard to prevalence of key behavioural risk factors both in Germany and in the EU as a whole. Doing so provides a foundation with which to develop and evaluate the effectiveness of evidence-based policy strategies and measures. Comparing results from Germany with the rest of the EU can help identify the potential for disease prevention and highlight health policy areas where action is needed so that health equity can be improved in Germany. The second wave of the European Health Interview Survey, which was carried out between 2013 and 2015 (EHIS 2), provides up-to-date, European-wide comparable data with which to describe and compare social differences in the frequency (prevalence) of behavioural risk factors among adults in Germany and the EU.

## 2. Methodology

### 2.1 Sample design and study implementation

As part of the European Health Interview Survey (EHIS), all EU Member States collect data on the health status, health care, health determinants and the socioeconomic situation of their populations (Info box 1). The survey targets people aged 15 or over who live in private households. The first EHIS wave (EHIS 1) was conducted between 2006 and 2009, but Member States were not obliged to participate in the study at this time. Data acquisition for the second EHIS wave (EHIS 2) took place between 2013 and 2015 in all 28 EU Member States. In order to ensure a high degree of harmonisation between the survey results from the various Member States, a handbook was provided with recommendations and guidelines on methodology and implementation, as well as a model questionnaire [7]. In Germany, EHIS forms part of the health monitoring undertaken at the Robert Koch Institute. EHIS 2 has been integrated into the German Health Update (GEDA 2014/2015-EHIS) [8, 9]. A detailed description of the methodology applied in GEDA 2014/2015-EHIS can be found in Lange et al. [9].

**Info box 1:**

**European Health Interview Survey (EHIS)**

The European Core Health Indicators (ECHI) were jointly developed by EU Member States and international organisations, taking into account scientific and health policy requirements. The indicators provide a framework in European health reporting for population-based health surveys and analyses, and health care provision at the European and national level. The European Health Interview Survey (EHIS) is a key element in this regard. The first EHIS wave (EHIS 1), which was not mandatory, was conducted between 2006 and 2009. 17 Member States and two non-EU countries participated in EHIS 1. Participation in the second wave of EHIS (EHIS 2), which was conducted between 2013 and 2015 in all EU Member States (as well as in Iceland, Norway and Turkey) was legally binding and is based on Commission Regulation (EU) No 141/2013 of 19 February 2013. It provides essential information about the ECHI indicators. In Germany, EHIS is carried out as part of health monitoring at the Robert Koch Institute. During the EHIS 2 survey period, the EU had 28 Member States.

Further information is available at: https://ec.europa.eu/eurostat/web/microdata/european-health-interview-survey

Each EU Member State chose a nationally representative sample for EHIS 2 based on data from population registers, censuses, housing registers or other statistical sources. Data acquisition was planned to last at least three months and include at least one autumn month (September to November). The average length of data collection across all EU Member States was eight months. A quality report was to be produced by each participating Member State following specific criteria. These reports provide detailed information about the methodology implemented by a respective country [10]. A more detailed description of the methodology applied in EHIS 2 is available in Hintzpeter et al. in this issue of the Journal of Health Monitoring [11]. In Germany, the survey used a two-stage stratified cluster sample that was randomly drawn from local population registers and was conducted between November 2014 and July 2015 [9].

### 2.2 Indicators

#### Smoking

Data on a participant’s smoking status were collected using the question ‘Do you smoke?’ The following answer categories were provided: ‘Yes, daily’, ‘Yes, occasionally’, ‘No, not any more’ and ‘I have never smoked’ [12]. Current smoking was defined as daily or occasional smoking.

#### Obesity

Body height and weight were determined using the following questions: ‘How tall are you without shoes?’ and ‘How much do you weigh without clothes and shoes?’ [12]. BMI was calculated using the formula: body weight in kilogrammes divided by height in metres squared. Obesity was defined as BMI ≥ 30kg/m^2^ in line with the classification used by the World Health Organization (WHO).

#### Physical activity

The duration of aerobic physical activity was determined using the EHIS-Physical Activity Questionnaire (EHIS-PAQ) [12-14]. The questionnaire covers physical activity conducted during leisure, work, or for transport. In drawing up the indicator, consideration was given to aerobic physical activity undertaken during leisure time as well as cycling for transportation. In line with WHO categories, low physical activity during leisure time was defined as aerobic physical activity amounting to less than 150 minutes per week.

#### Alcohol intake

Data were collected on heavy episodic drinking of 60g or more of pure alcohol on one occasion using the following question: ‘In the past 12 months, how often have you had 6 or more drinks containing alcohol on one occasion?’ [12]. Heavy episodic drinking was defined as the consumption of six or more alcoholic drinks on one occasion at least once a month.

#### Fruit and vegetable intake

Data on the participants’ fruit and vegetable intake were collected separately for fruit and vegetables before being combined into one variable: ‘How often do you eat fruits/vegetables or salad, including freshly squeezed fruit/vegetable juices?’ [12]. Non-daily fruit or vegetable intake was defined as the consumption of less than one portion of fruit or vegetables per day.

#### Education

Participants’ education level was assessed according to the 2011 version of the International Standard Classification of Education (ISCED) [15]. The ISCED system takes both educational and vocational qualifications into account and enables international comparative analyses to be undertaken of groups of people with differing levels of education in countries with different educational systems. For the purpose of this article, ISCED levels 0 to 2 were merged into a low education group, ISCED levels 3 to 4 into a medium education group, and ISCED levels 5 to 8 into a high education group.

### 2.3 Statistical analyses

The analyses are based on data from a total of 217,215 participants (116,895 women, 100,320 men) aged between 25 and 69 from EU Member States (Table 1). The age limitation was put in place to reduce potential bias from younger cohorts who are still to gain educational qualifications and from older cohorts who received their education prior to educational expansion, i.e. the increased participation of post-war generations in secondary and tertiary education. The following numbers of participants were excluded as they lacked data for the respective indicators: 3,313 participants for smoking, 8,102 for obesity, 20,836 for physical activity (no data were available from the Netherlands or Belgium), 41,007 for heavy episodic drinking (no data were available from France, the Netherlands or Italy) and 5,751 for fruit or vegetable intake.

**Info box 2:**

**Calculating and interpreting absolute and relative differences using the Slope Index of Inequality (SII) and the Relative Index of Inequality (RII)**

The SII and RII are regression-based measures that take into account the entire distribution of socioeconomic variables such as education and the size of socioeconomic groups [17, 18]. In the analyses undertaken for this article, linear probability models were used to calculate the SII and log-binomial models were used to calculate the RII. This involved converting the education variable to a metric scale ranging from 0 (the highest level of education) to 1 (the lowest level of education) by means of ridit analysis [21]. Education was then included as an independent variable in the regression models [22]. The resulting regression coefficients indicate SII or RII, depending on the model. Age-standardisation was applied during the calculation of both indices. SII is a measure of the prevalence difference (absolute inequality), whereas RII indicates the prevalence ratio (relative inequality) between individuals with the lowest and highest level of education. For example, an SII of 0.15 indicates that a 15 percentage-point difference exists between people at the bottom of the educational scale and those at the top. An SII of 0.00 means that no prevalence difference exists between these individuals. An RII of 2.00 indicates that people at the very bottom of the educational scale are twice as likely to face a particular risk factor compared to those at the very top of the educational scale. An RII of 1.00 indicates that no relative risk difference exists between these individuals.

The analyses were performed with a weighting factor to ensure that each EU Member State was considered in proportion to its population size. In contrast to the analyses undertaken for GEDA 2014/2015-EHIS for Germany [9], education was not taken into account during weighting for the European comparison; this follows current recommendations of the Statistical Office of the European Union (Eurostat). The household indicator variable was used as the cluster variable in the following analyses. All analyses were performed using Stata 15.1.

Prevalences with 95% confidence intervals (95% CI) stratified by education and sex were estimated for each of the observed behavioural risk factors. Prevalences are estimates, the precision of which can be assessed through their CI; wide CI indicate that a particular outcome has a greater level of statistical uncertainty. Prevalences were calculated for all EU Member States as a whole, and separately for Germany. Prevalence differences occur in cases where the CI of prevalence estimates do not overlap. In order to provide for an adequate comparison of prevalence estimates, direct age standardisation was applied. In each case, the age structures of the samples in Germany and other EU Member States were adjusted to reflect the 2013 revision of the European Standard Population (ESP) [16]. In the analyses that were stratified by education, age standardisation was also applied to education groups within each country. Age standardisation provides for a direct comparison of prevalence estimates as it corrects for differences in the age structure of a population or subpopulation. The Slope Index of Inequality (SII) and the Relative Index of Inequality (RII) were used to examine the extent of absolute and relative educational differences in the prevalence of behavioural risk factors [17, 18]. Whereas the SII quantifies the extent of absolute prevalence differences between education groups (absolute inequality), the RII provides a measure of the extent of the prevalence ratio (relative inequality) that exists between education groups (Info box 2). As studies that merely use absolute or relative differences can produce one-sided, selective conclusions, the literature recommends calculating both absolute and relative measures of inequality [19, 20]. This is particularly important when target variables appear on different overall levels, as is the case with this analysis of the prevalence of behavioural risk factors. SII and RII were calculated for all EU Member States together (including Germany) and separately for each Member State in order to provide for an analysis of where the values for Germany stand in terms of the range of results gained from the various Member States.

In order to provide a clear overview of the indicators, the individual values that were calculated for each of the 28 EU Member States are not set out in Figure 2 or Figure 3. Instead, the figures provide the lowest and highest values from the EU Member States for which data are available, the EU average for the Member States under study, and the prevalence for Germany.

## 3. Results

Data from EHIS 2 show that low physical activity in leisure time and non-daily fruit or vegetable consumption are among the most prevalent risk factors for women and men aged between 25 and 69. This applies both to the population living in Germany and to the EU average (Table 2). A comparison of the age-standardised prevalence of risk factors in Germany with EU averages shows that the distribution of heavy episodic drinking and non-daily fruit or vegetable intake among women and men in Germany is well above the EU average. Obesity prevalence among men in Germany also slightly exceeds the average EU level. The prevalence of smoking, on the other hand, is lower among men in Germany than the average across the EU. Obesity and smoking prevalence among women in Germany are at the same level as the EU average. Low physical activity in leisure time, on the other hand, is less common among women and men in Germany than the EU average.

With the exception of low physical activity during leisure time, a comparison of women’s and men’s age-standardised prevalences shows that the risk factors under consideration are more common among men than women. This is particularly the case with regard to heavy episodic drinking and non-daily fruit or vegetable intake and applies both to Germany as well as the EU average. No differences were found between the sexes with regard to the prevalence of low physical activity in Germany, but the prevalence is higher among women than men on average throughout the EU.

**Figure 1:**
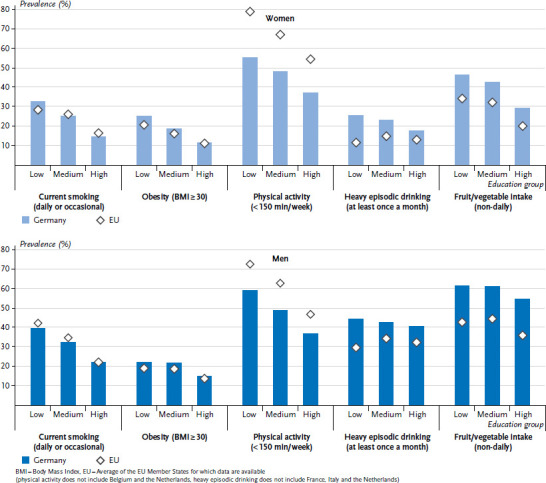
Age-standardised prevalence of behavioural risk factors by sex and education level Source: EHIS 2 (2013-2015)

When the results are differentiated according to education, significant differences become evident in the distribution of behavioural risk factors between education groups. In Germany, the lower the education level, the higher the age-standardised prevalence of smoking, obesity, low physical activity, and non-daily fruit or vegetable intake (Figure 1). This also reflects the EU average. However, the educational gradient for non-daily fruit or vegetable intake is not as pronounced for men as it is for women. The EU average prevalence of age-standardised heavy episodic drinking poses an exception. No consistent educational gradient was identified for the EU average among women or men. However, an educational gradient was identified for Germany, particularly among women: women from lower education groups show a higher prevalence of heavy episodic drinking.

**Figure 2:**
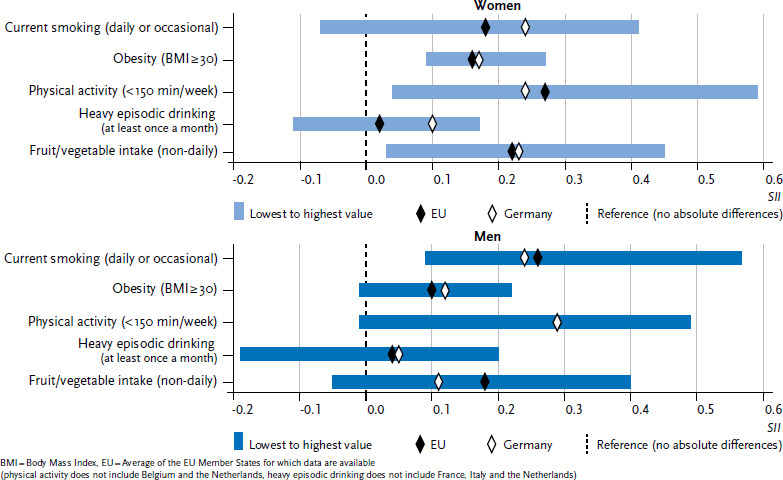
Absolute educational differences (SII) in the prevalence of behavioural risk factors (age-standardised) by sex Source: EHIS 2 (2013-2015)

**Figure 3:**
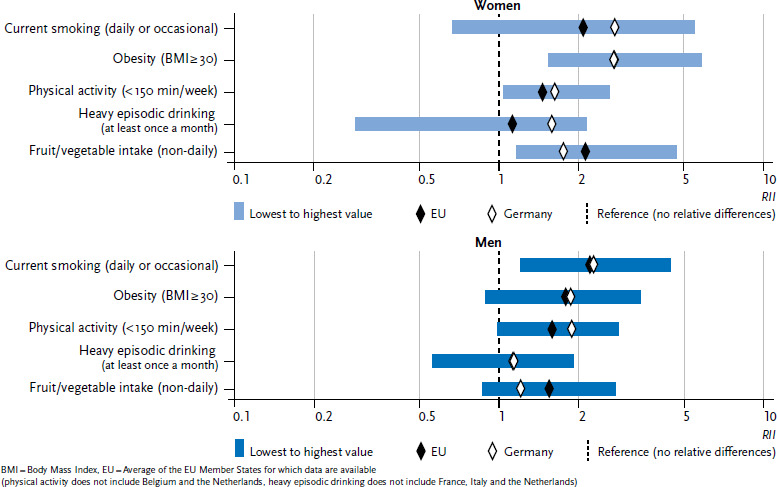
Relative educational differences (RII) in the prevalence of behavioural risk factors (age-standardised) by sex Source: EHIS 2 (2013-2015)

More detailed analyses not only reveal whether educational differences exist in the prevalence of behavioural risk factors, but also how stark these differences are in Germany compared to the EU average. Figure 2 shows the extent of absolute differences in education (the difference in prevalence between persons with lowest and highest education status) whereas Figure 3 shows the extent of relative education differences (the prevalence ratio between persons with lowest and highest educational status) for each of the five risk factors. The diamonds in both figures mark the SII or RII for Germany (white) and the EU average (black). The blue bars illustrate the range between the EU Member State with the highest and lowest value.

Whereas absolute and relative educational differences for smoking among women in Germany are greater than the EU average, the differences among men in Germany are very close to the EU average. Educational differences in obesity prevalence in Germany are roughly the same as the EU average for women and men in both absolute and relative terms. No significant educational differences were identified between Germany and the EU average for low physical activity in leisure time. No differences between Germany and the EU average were found for men in terms of the extent of educational differences in heavy episodic drinking. However, educational differences for heavy episodic drinking among women in Germany are greater than the EU average. Educational differences in non-daily fruit or vegetable intake in Germany are lower than the average across the EU. This is true for relative differences between the sexes and is predominantly the case among men with regard to absolute differences. Strikingly, an analysis of the extent of educational differences in the prevalence of behavioural risk factors across the EU (the blue bars in Figure 2 and Figure 3) generally places Germany in the middle range compared to other EU Member States.

## 4. Discussion

### 4.1 Main results

In Germany and most other EU Member States, behavioural risk factors are more prevalent among lower education groups than higher education groups. Whereas differences in smoking prevalence among women in Germany are greater than the EU average, educational differences in obesity prevalence in Germany correspond to the average EU level. Similarly, although educational differences in heavy episodic drinking among women in Germany are greater than the EU average, educational differences in non-daily fruit or vegetable intake in Germany are lower than the average across all EU Member States. To the best of our knowledge, this study represents the first population-based analysis of absolute and relative educational differences of behavioural risk factors among adults that compares Germany with the EU average.

### 4.2 Interpretation and discussion of the results

Recent reviews indicate that the vast majority of published studies have found significant links between education and mortality [23, 24]. Galamar et al. [24] highlight two causal paths to explain this association. First, greater educational attainment leads to higher incomes and wealth over a person’s lifetime, which in turn provides them with better access to health resources, and leads to healthier behaviour and patterns of consumption. Second, greater educational attainment fosters the development of health-promoting skills such as health literacy and access to quality health services [24]. International findings also demonstrate that educational differences in health behaviour (or behavioural risk factors such as smoking, physical inactivity and obesity) significantly contribute to educational differences in mortality [25-28]. As such, it is essential to consider the fact that patterns of behaviour are embedded within specific conditions, in other words, they are shaped, or at least influenced, by people’s living and working conditions, as well as the associated psychosocial factors [28, 29].

#### Current smoking

Tobacco use is the most common single cause of premature mortality in the EU. According to the European Commission, nearly 700,000 EU citizens die as a result of smoking every year [30]. Women in Germany smoke as frequently as the European average, with men in Germany doing so less frequently. This finding corroborates the results of other comparative European studies [31]. One finding that is particularly striking, however, is the fact that the educational gradient for smoking among women in Germany is much more marked than the European average. The educational gradient for tobacco use among women in southern European countries, for example, is much smaller [32, 33]. It is possible that this is because Germany and other northern and central European countries are currently in a later phase of the ‘smoking epidemic’ [33, 34, 35]: smoking is said to initially become widespread among privileged sections of the population, especially among men. Over time, men from lower status groups adopt the habit, followed by high-status women. Eventually, smoking also becomes common among women with a low socioeconomic status [33, 36].

Trend analyses demonstrate that the educational gradient in smoking behaviour in Germany has not only remained undiminished since the turn of the millennium, but also that it has actually increased slightly among men [37]. Moreover, this has occurred despite the numerous tobacco control measures that have been introduced during this period [38]. This demonstrates that preventive measures with the potential to further reduce smoking need not necessarily be associated with a decline in social inequality [39]. When establishing new measures, therefore, it is important to ensure that they do not exacerbate existing socioeconomic-related health inequalities and cause intervention-generated inequality [40]. Despite the falling prevalence in smoking [35] and a smoking rate that is average by European standards, there is still considerable room for improvement in Germany with regard to smoking prevention policy: a European comparison of different areas of tobacco control places Germany second from bottom [41].

#### Obesity

People who are obese are more likely to suffer from chronic diseases such as type 2 diabetes, cancer or cardiovascular disease and generally have a lower life expectancy than those with a normal weight [42, 43]. The prevalence of obesity has increased dramatically worldwide in recent decades [44]. This also poses a major challenge – not least for the health sector – that looks set to continue for years to come as obesity prevalence is predicted to rise in the future [44]. Although obesity prevalence is higher in Germany than the EU average, educational differences in obesity are similar to the EU average. There is a clear gradual reduction in obesity levels associated with higher education level. Previous surveys have also identified a clear gradient for obesity prevalence and socioeconomic status among adults in Germany [45]. A recent study analysed temporal changes in educational differences in obesity between 1990 and 2010 in 15 European countries [46]. Overall, the study observed an increase in obesity prevalence over time but the extent of this increase varied between the countries under study. Moreover, this increase was greater in absolute terms among people with a low education level. There has, however, been no increase in relative educational differences [46]. These differences associated with socioeconomic or education level are primarily due to long-term differences in nutrition and physical activity. Although it is very difficult for population studies to describe this situation precisely, it is reflected, among other factors, in the observed differences in physical activity as well as fruit and vegetable intake.

#### Low physical activity during leisure time

People who follow the recommended minimum of at least 150 minutes of aerobic physical activity per week have a 40% lower risk of premature mortality compared to people who are physically inactive [47]. In Germany, low physical activity in leisure time (less than 2.5 hours a week) is much less common among women and men than across the EU average. However, pronounced educational differences do exist in the prevalence of low physical activity in leisure time both in Germany and the EU average. These differences can be mapped as a gradient that demonstrates the lower the education level, the higher the prevalence of low physical activity. This observation also reflects the results of an analysis based on the pooled data set used by the 2014 European Social Survey which were calculated for 21 European countries. The results identified a gradient with regard to physical activity and education on at least three days a week to the detriment of lower education groups [6].

The relative educational differences in the prevalence of low physical activity in leisure time are similar to the EU average for women in Germany and slightly above the EU average for men. The slightly greater educational differences among men in Germany compared to the EU average could be due to Germany’s relatively large service sector as it contributes to high levels of physical inactivity at work among men with a high education level [48, 49]. Men with a high education level, however, may compensate for work-related inactivity with greater physical activity during leisure time [50]. By contrast, men with a medium or low education level are more active at work and engage less frequently in physical activities during leisure time [48, 49].

A recent trend analysis of relative educational differences in sporting activity among adults in Germany points out that differences increased after the turn of the millennium. These differences can be explained by a strong increase in the prevalence of physical activity among adults with high education levels compared to those with a low education status [51]. In Germany as well as in the EU as a whole, additional evidence-based measures are needed to promote aerobic physical activity at the population level, reduce social differences in aerobic physical activity, and counteract the development of health inequalities [52]. The Global Activity Plan on Physical Activity and Health 2018-2030 recommends a multi-component approach that focuses on the systemic, social, environmental and individual levels [53]. The proposed measures are aimed at reducing the prevalence of inadequate levels of physical activity by 15% (compared to 2010 levels) by 2030 [53].

#### Heavy episodic drinking

In Germany, heavy episodic drinking – in other words, drinking 60g or more of pure alcohol on one occasion – is significantly more widespread among women and men than the European average [54]. Heavy episodic drinking is a particularly risky form of drinking which, in addition to the long-term effects of excessive alcohol consumption, such as alcohol dependency and organ damage, can also result in acute damage due to alcohol intoxication, cause people to injure themselves or others, and even lead to violence [55].

A social gradient for heavy episodic drinking exists among women in Germany that is not found in the EU average. Heavy episodic drinking is less prevalent among women in Germany with a high education level than among women with a low education level. However, there is a large range of differences between countries that even includes partially reversed gradient in some Member States. Another European study that covered heavy episodic drinking found clear educational differences between countries with regard to alcohol consumption [6]. However, it only identified a slight gradient for Germany and this was not associated with a particular sex.

In contrast to heavy episodic drinking, a number of German studies on risky alcohol consumption, defined as more than 10g (for women) or more than 20g (for men) of pure alcohol in one day, found no gradient associated with men’s social or education level, and a reversed gradient in this case among women [56-58].

Harmful alcohol consumption is not associated with the same consequences in all status groups. Similar levels of alcohol intake cause greater damage to the health of disadvantaged population groups than privileged groups; this difference is referred to as the alcohol harm paradox [59]. Alcohol intake is more likely to cause problems for women and men with low education level. These individuals are also often less able to compensate for the related social and health difficulties [60]. This is particularly problematic because heavy episodic drinking appears to be particularly widespread among women in groups with low education level. In Germany, far fewer regulatory measures have been introduced to limit the population’s alcohol consumption than in many other EU countries [61]. For example, taxes on alcoholic beverages in Germany are much lower than the EU average [61].

#### Non-daily fruit and vegetable intake

Non-daily fruit or vegetable intake is much more common in Germany than the EU average. However, Germany also has a less pronounced educational gradient in this regard. A low fruit and vegetable intake is a risk factor associated with coronary heart disease, hypertension and stroke [62]. The Global Burden of Disease Study estimates that in 2017, approximately two million deaths worldwide were associated with low fruit intake and approximately 1.5 million deaths were associated with low vegetable intake [63]. For this reason, among others, recommendations encourage people to eat fruit and vegetables on a daily basis. However, the German Health Interview and Examination Survey for Adults (DEGS1, 2008-2011) shows that many adults in Germany, especially adults with a low socioeconomic status, do not follow this recommendation [64]. Data from EHIS 2 were used to calculate the percentage of adults that consume at least five portions of fruit and vegetables per day in each EU Member State stratified by education group [65]. The resulting indicator paints a picture that corroborates the results of this study: both the percentage in Germany and the educational differences for Germany are lower than the EU average [65]. Further comparable European-wide studies have mostly found that people with a higher education level have a greater or more frequent fruit and vegetable intake than people with a lower education level, with the exception of some southern and eastern European countries [6, 65-67]. However, these studies also found considerable variations in educational differences between the EU states [6, 65-67]. As such, significant differences exist in fruit and vegetable intake within the EU and these differences are influenced by many aspects, including culture. Measures to increase fruit and vegetable intake, therefore, should take educational differences and regional influences into account.

### 4.3 Strengths and limitations

EHIS 2 has a number of strengths. The study is based on a large number of cases as more than 200,000 people participated throughout the EU. The study also expected high standards to be put in place for the sampling framework drawn up by each country. As such, EHIS 2 enables representative findings to be made for individual countries and the EU as a whole. The high degree of standardisation and harmonisation of the EHIS questionnaire and the resulting data, which enable individual countries to be compared with the EU as a whole, should also be stressed in this context [10, 68]. However, when interpreting the results of the study, it is important to note that the data from Germany are also included in the EU average. At the same time, significant differences not only exist in the prevalence of behavioural risk factors within the EU, but also between countries when it comes to factors such as economic performance and structure, as well as a country’s welfare system, social stratification and degree of urbanisation. In addition, as the data collected for the study were self-reported, they are subject to unavoidable limitations such as reporting bias, recall bias, and the provision of socially desirable responses [69, 70]. Data collection periods did not always cover an entire year nor were they always conducted for the same length of time in each country. As such, seasonal variations, which do affect some of the behavioural risk factors under study, may have influenced the results. The cross-sectional study design of EHIS means that no causal inferences can be deduced from the observed associations between education level and behavioural risk factors. Finally, it is not altogether impossible that the results only provide limited generalizability due to sample bias or the unavailability of data for specific indicators in individual countries.

### 4.4 Conclusion

Compared to the EU average, Germany appears in the middle range of a comparison of relative educational differences for five behavioural risk factors. Relative educational differences in current smoking, heavy episodic drinking among women and low physical activity in leisure time among men are more pronounced in Germany than in the EU average. However, the relative educational differences identified in fruit and vegetable intake among women and men are less distinct in Germany than in the EU average. The observed educational gradient, whereby people with a lower education level have a higher prevalence of behavioural risk factors, is consistent with the significant gap in life expectancy that has been identified between lower and higher education groups in Germany and the EU [2, 71]. Against this backdrop, non-governmental organisations are vehemently demanding the implementation of health policy measures aimed at improving health equity. Such measures should focus on conditional factors and follow the ‘health in all policy fields’ approach. In other words, they should be implemented at the systemic, social, environmental and meta-population levels, and particularly prioritise disadvantaged groups so as to make ‘healthy choices easy choices’ [72, 73].

## Key statements

- Marked educational differences exist in behavioural risk factors in Germany and most other EU Member States.
- Educational differences in smoking among women in Germany are greater than the EU average.
- Educational differences in the prevalence of obesity and low physical activity in Germany correspond to the EU average.
- Educational differences in heavy episodic drinking among women in Germany are greater than the EU average.
- Educational differences in non-daily fruit or vegetable intake in Germany are smaller than the EU average.

## Figures and Tables

**Table 1:** Characteristics of the study population by sex (n=116,895 women, n=100,320 men) Source: EHIS 2 (2013-2015)

	Women		Men	
	%	n	%	n
**Education level**				
Low education group	24.9	29,922	23.6	25,030
Medium education group	43.6	50,147	46.1	46,764
High education group	31.5	36,129	30.3	27,900
**Risk factor**				
Current smoking (daily or occasional)	22.9	25,676	32.4	32,797
Obesity (BMI ≥ 30)	15.9	18,919	17.1	17,299
Physical activity (< 150 min/week)^1^	66.4	71,723	60.2	56,380
Heavy episodic drinking (at least once a month)^2^	13.3	11,185	32.5	25,066
Fruit/vegetable intake (non-daily)	28.5	32,661	41.1	39,255
**Countries**				
Austria	1.7	7,147	1.7	5,632
Belgium	2.0	3,353	2.0	3,180
Bulgaria	1.5	2,343	1.5	2,245
Croatia	0.8	1,925	0.9	1,823
Cyprus	0.2	1,848	0.2	1,671
Czech Republic	2.2	2,523	2.2	1,990
Denmark	1.1	2,202	1.1	1,812
Estonia	0.3	2,155	0.3	1,697
Finland	1.0	2,445	1.1	1,920
France	12.2	5,888	11.7	5,314
Germany	16.0	9,732	16.5	7,805
Greece	2.2	3,329	2.1	2,312
Hungary	2.0	2,195	2.0	1,937
Ireland	0.9	4,426	0.9	3644
Italy	12.2	9,036	12.2	8,597
Latvia	0.4	2,707	0.4	2,050
Lithuania	0.6	2,093	0.5	1,404
Luxembourg	0.1	1,690	0.1	1,415
Malta	0.1	1,549	0.1	1,396
Netherlands	3.2	2,821	3.3	2653
Poland	8.1	9,513	7.9	7,981
Portugal	2.1	6,927	2.0	5,691
Romania	4.0	6,030	4.1	5,690
Slovakia	1.1	2,207	1.1	1,853
Slovenia	0.4	2,386	0.4	2,028
Spain	9.5	8511	9.6	7,892
Sweden	1.8	2,070	1.9	2,237
United Kingdom	12.2	7,844	12.2	6,451

% = weighted proportion, n = unweighted number of participants

^1^ Excluding Belgium and the Netherlands (no data available)

^2^ Excluding France, Italy and the Netherlands (no data available)

**Table 2:** Age-standardised prevalence of behavioural risk factors by sex Source: EHIS 2 (2013-2015)

	Women		Men	
	%	(95% CI)	%	(95% CI)
**Current smoking** **(daily or occasional)**				
Germany	22.1	(21.2-23.1)	28.4	(27.2-29.5)
EU	22.9	(22.6-23.3)	32.3	(31.9-32.7)
**Obesity (BMI ≥ 30)**				
Germany	16.6	(15.8-17.5)	18.6	(17.6-19.5)
EU	15.8	(15.5-16.1)	17.2	(16.9-17.5)
**Physical activity** **(< 150 min/week)**				
Germany	45.1	(43.9-46.2)	44.4	(43.2-45.7)
EU	66.3	(65.9-66.7)	60.3	(59.8-60.7)
**Heavy episodic drinking** **(at least once a month)**				
Germany	21.7	(20.8-22.7)	41.8	(40.6-43.1)
EU	13.4	(13.1-13.7)	32.5	(32.0-33.0)
**Fruit/vegetable intake (non-daily)**				
Germany	38.4	(37.3-39.5)	58.3	(57.0-59.5)
EU	28.6	(28.2-28.9)	41.0	(40.6-41.5)

CI=Confidence interval, EU=Average of EU Member States for which data are available (physical activity does not include Belgium and the Netherlands; heavy episodic drinking does not include France, Italy and the Netherlands)
